# Primary Vaginal Melanoma or Clear Cell Sarcoma: A Difficult Differential Diagnosis

**DOI:** 10.7759/cureus.83443

**Published:** 2025-05-04

**Authors:** Dimitrios Panagiotopoulos, Dimos Sioutis, Charikleia Papageorgiou, Vaia G Sarli, Chrisi Christodoulaki, Theofanakis Charalampos, Nektarios I Koufopoulos, Vasileia Damaskou, Periklis Panagopoulos, Nikolaos Machairiotis

**Affiliations:** 1 Third Department of Obstetrics and Gynecology, Attikon University Hospital, National and Kapodistrian University of Athens, Athens, GRC; 2 Second Department of Pathology, Attikon University Hospital, National and Kapodistrian University of Athens, Athens, GRC

**Keywords:** biopsy, immunohistochemistry, melanoma, sarcoma, vaginal

## Abstract

Lower genital tract lesions are a frequent reason for gynecological consultation, and primary vaginal melanoma is an uncommon but aggressive form of malignancy. It primarily strikes in postmenopausal women and is often detected at an advanced stage, accounting for its poor prognosis. A 47-year-old Caucasian female patient was referred to our institution due to abnormal vaginal bleeding and a palpable lesion near the urethra, and subsequently, we present a case of primary vaginal melanoma. Histopathological and immunohistochemical studies confirmed the diagnosis of melanoma and differentiated it from clear cell sarcoma. Imaging showed multiple lung metastases, and the patient was restaged as stage IV vaginal melanoma. The patient was treated with anti-PD-1 agent nivolumab and anti-CTLA-4 agent ipilimumab, and after that, she showed a good clinical response. This case illustrates some of the difficulties in wade through the diagnosis and treatment of vaginal melanoma, as well as a powerful argument for obtaining early definitive biopsy and diagnosis. We discuss the treatment options, including surgical excision, radiation therapy, and immunotherapy, emphasizing the poor prognosis and low survival rates among patients diagnosed with an advanced stage.

## Introduction

Lesions of the lower genital tract are a common reason for admission at a gynecology clinic. Most of them arise from the epithelial lining of the vulva, vagina, or cervix. Primary vaginal melanoma is a rare condition, which can spread to distal organs, usually affecting postmenopausal patients. The patients are often diagnosed at an advanced stage [1]. The treatment includes radical surgery, radiation, and chemotherapy with immunotherapy. The prognosis is poor in the majority of cases. In this manuscript, we report a case of primary vaginal melanoma and discuss the differential diagnosis with clear cell sarcoma, and we review the literature.

## Case presentation

**Figure 1:**
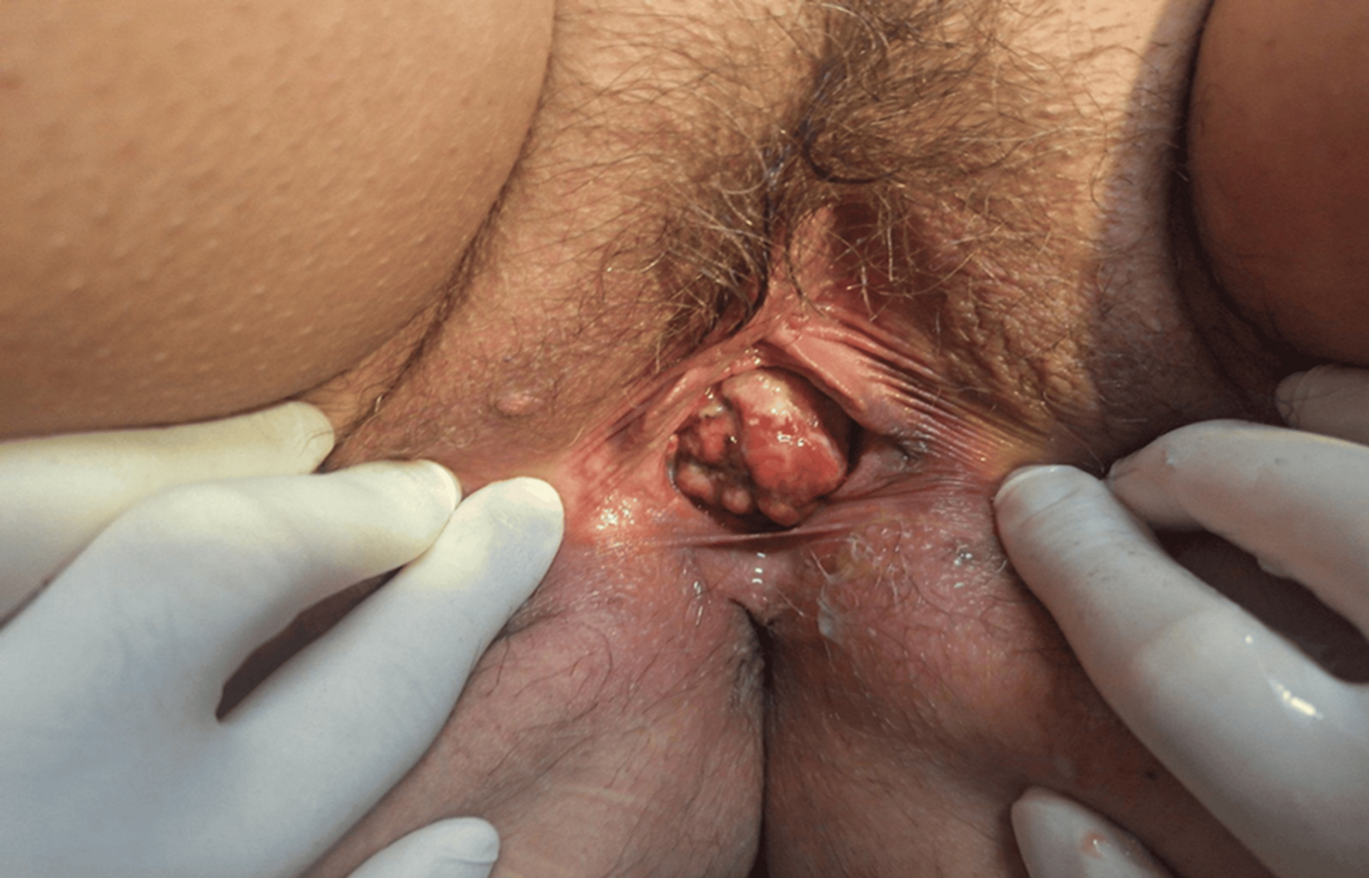
Vaginal melanoma arising from the lower third of the vagina, close to the urethra.

**Figure 2:**
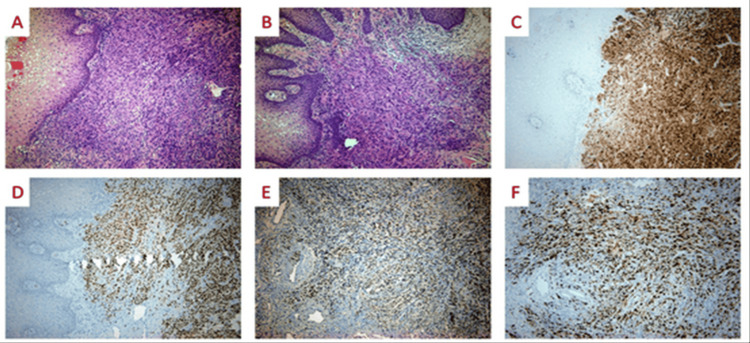
(A-F) Histopathological examination.

We present a case of vaginal melanoma in a 47-year-old, para 2, Caucasian woman who was referred for abnormal vaginal bleeding and persistent palpable lesion close to the urethra. Her last pap smear was normal two years ago, and her medical history was free. At the gynecological examination, a raised, friable, and hemorrhagic lesion on the anterior vaginal wall with a maximum diameter of 40 mm was noticed (Figure 1). Although the lesion was close to the urethra, neither urinary obstruction nor fistula occurred. On bimanual examination, the pelvis was free of any palpable disease. A digital exam of the rectum was negative for solid mass or blood. There were no palpable inguinal lymph nodes. Urine analysis showed no red blood cells or hemoglobin in the urine. Two punch biopsies were taken at the office. Hemostasis was performed with Monsel’s solution. The histological examination of the biopsy specimen showed a subepidermal spindle cell neoplasm without an epidermal component (Figures 2A, 2B). Immunohistochemically, tumor cells were positive for Melan A (Figure 2C) and SOX-10 (Figure 2D), displaying also focal weak positivity for PRAME (Figure 2E). Ki67 stained most neoplastic cells (Figure 2F).

Giant cells were not present. Melanin pigment was not identified. The histological diagnosis was vaginal malignant neoplasm morphologically and immunohistochemically consistent with melanoma versus clear cell sarcoma. The molecular study of the tumor was negative for the EWSR1-ATF1/CREB1 fusion. Given all the above facts, the tumor was considered malignant melanoma.

For staging, an MRI of the abdomen was performed, which showed a primary lesion on the vaginal wall at 46 x 25 x 20mm with no other enlarged lymph nodes or metastases in the parenchymal organs of the abdomen. The CT scan showed no metastases in the central nervous system. Unfortunately, a chest CT scan revealed multiple lung metastases. The patient was staged as a stage IV vaginal melanoma.

She was admitted to the oncologic clinic and treated with the anti-PD-1 agent nivolumab and anti-CTLA-4 agent ipilimumab. After the second session of monoclonal antibodies, the patient reported diarrhea, which was managed successfully with per os methylprednisolone. The patient is in good clinical status nine months after the initial diagnosis. She does not report any episode of vaginal bleeding, shortness of breath, hemoptysis, dizziness, or headache. On clinical examination, the vaginal lesion has the same size without obstructing the urethra.

## Discussion

Primary vaginal malignancies are rare, accounting for only 2% of female genital tract neoplasms. Vaginal melanomas are 5% of all vaginal malignancies [1]. The most common site of occurrence is the lower third of the vagina. The lesions are usually exophytic, polypoid, and ulcerated. Ten percent of them are amelanotic, as in our case. The main differential diagnosis of melanoma in our case was clear cell sarcoma. Melanoma shares similar histologic and immunohistochemical features (both are positive for melanocytic markers such as S100, SOX-10, HMB45, MITF, and Melan A with clear cell sarcoma). On the other hand, melanoma is more common in the elderly, shows usually an epidermal component, lacks the giant cells seen in clear cell sarcoma, and on the molecular level lacks the EWSR1-ATF1/CREB1 mutation. Vaginal bleeding and discharge, postcoital bleeding, and palpable lesion are the most common symptoms. Common sites of metastasis are the liver, lungs, brain, and bones [1]. Due to the high rate of metastatic spread at the time of diagnosis, the patient's staging includes a chest and abdomen CT scan or a whole-body fluorodeoxyglucose (FDG) PET CT and brain MRI. Tumor size less than 3 cm appears to be the only significant prognostic factor of survival in the early stages. Due to its rarity, there is no consensus or guidelines on the treatment of vaginal melanomas. The treatment options follow the standard practice for melanomas. If feasible, surgical excision in the early stages increases overall survival. Wide local excision is preferred, given that radical surgical approaches such as radical vulvovaginectomy and pelvic exenteration have a high peri- and postoperative morbidity without a significant difference in survival rate. Recommended excision margins follow the rule of 1 cm free margins. The role of lymphadenectomy is not clear in vaginal melanomas. Vaginal melanomas show a low rate of lymph node metastasis. The vaginal lymph drainage is complex-the upper third of the vagina drains to external iliac nodes, the middle third drains to common and external, and the lower third drains to superficial inguinal and perirectal lymph nodes [2]. PET CT may be beneficial in the identification of affected lymph nodes. While complete lymphadenectomy does not seem to improve a patient's outcome, sentinel lymph node biopsy appears to be a practice that aids in the information of a patient's node status. Radiation therapy may be used for tumor reduction to avoid an extensive surgical excision. Otherwise, radiotherapy provides local control in patients with unresectable disease [2]. Chemotherapy is controversial for the postoperative treatment of vaginal melanoma. There may be a place for non-surgical candidates or patients with recurrence [3]. Most of the cases are characterized by PD-1/PDL-1 expression. Therefore, immune checkpoint inhibitors such as nivolumab, pembrolizumab, and ipilimumab are used as first-line treatment for metastatic or unresectable melanoma [3]. The prognosis of these patients is generally poor. The five-year survival rate for patients with vaginal melanoma is estimated at 5%-27%. Tumor size (>3 cm) is the most significant factor in the early stages, whereas lymph node involvement is associated with decreased survival [2].

## Conclusions

Vaginal melanoma is a rare and aggressive neoplasia of the lower female genital tract. Additionally, patients are often diagnosed at an advanced stage. These reasons lead to a poor prognosis for these patients. A biopsy of any lesion with suspicious characteristics needs to be taken before any surgical or medical management decision. Wide local excision, if feasible, is the best option for controlling the disease. Radiation therapy and immunotherapy are the treatment options for metastatic or unresectable lesions.
